# Rocking pneumonia

**DOI:** 10.1186/s41479-017-0043-0

**Published:** 2017-12-15

**Authors:** Ger T. Rijkers, Maria Rodriguez Gomez

**Affiliations:** Science Department, University College Roosevelt, P.O. Box 94, 4330 AB Middelburg, The Netherlands

**Keywords:** Rocking pneumonia, Lyrics, Symptoms, Comorbidity, Treatment

## Abstract

Ever since Chuck Berry coined the term “rocking pneumonia” in his 1956 song “Roll over Beethoven”, pneumonia has been mentioned frequently in modern blues and rock songs. We analyzed the lyrics of these songs to examine how various elements of pneumonia have been represented in popular music, specifically the cause of pneumonia, the risk groups, comorbidity (such as the boogie woogie flu), the clinical symptoms, and treatment and outcome. Up to this day, songwriters suggest that pneumonia is caused mainly by the cold and rain and that treatment is hardly possible, aside from a shot of rhythm and blues.

In 1926, Virginia Woolf published an essay “On being ill”, in which she wondered why “illness has not taken its place with love, battle and jealousy among the prime themes of literature” [1]. Illness has not been a prime theme of literature, or music lyrics, for that matter, yet it has a significant impact on the human condition. Pondering this oversight, Woolf specifically called for odes to pneumonia, novels devoted to influenza, epic poems to typhoid, and lyrics to toothache.

Now, more than 90 years after Virginia Woolf’s essay, this question regarding illness’s representation in literature can be addressed with modern search techniques. The lyrics database www.lyrics.com considers itself “the web’s largest resource for music, songs and lyrics” and it is the latter functionality that was used for this research into how illness—specifically pneumonia—has been represented in music lyrics.

Search results revealed that the word ‘pneumonia’ is found in 239 song lyrics, which is a rather modest number when compared to the word ‘jealous’, which is found in 3879 lyrics, and ‘love’, which found in 324,016 lyrics. Pneumonia, however, clearly outperforms the other diseases suggested by Virginia Woolf: (novels devoted to) influenza, 29; (epic poems to) typhoid, 11; and (lyrics to) toothache, 73. In her essay she suggested “love and jealousy are states that spark the language but that the sufferer has the pain in his head but the language runs dry*”* [1]. This suggestion of the experience of pain overruling the ability to of language to adequately describe it warrants an analysis of the language that is used in songs about pneumonia.

“I got a rocking pneumonia, I need a shot of rhythm and blues” [2] is a famous line from the song “Roll over Beethoven”, a Chuck Berry original (1956) which has been covered by many others, including The Beatles (1963) and Electric Light Orchestra (1973). Briefly, to highlight one way in which an understanding of pneumonia in lyrics can be utilized in an academic setting, Chuck Berry’s lyric about pneumonia could be used in a creative multiple-choice question in an immunology or microbiology exam, structured as follows:

*Streptococcus pneumoniae* can cause a) otitis media, b) meningitis, c) pneumonia and d) bacteremia. Which one of these four manifestations of a pneumococcal infection did Chuck Berry have for which he needed a shot of rhythm and blues?

We have put this question to bachelor students at the University College Roosevelt (The Netherlands) and the frequency distribution of the answers is an almost perfect 25% for each possibility, so the general knowledge of university students of classical rock and roll song texts appears to be limited. It can only be hoped that their knowledge of the pneumococcus is better.

Chuck Berry’s “Roll over Beethoven” was recorded in 1956 (Fig. 1). The top position the song reached in the Billboard Hot 100 was a modest 29th place. About a year later, “A Rockin’ Pneumonia and the Boogie Woogie Flu” by Huey Smith and his Clowns made its first appearance on Billboard’s rhythm and blues chart. In his biography, Huey Smith admits that he had heard Chuck Berry sing “I got the rocking pneumonia, I need a shot of rhythm and blues” [3]. He was inspired to use this term and added “the boogie woogie flu” himself.

**Fig. 1:**
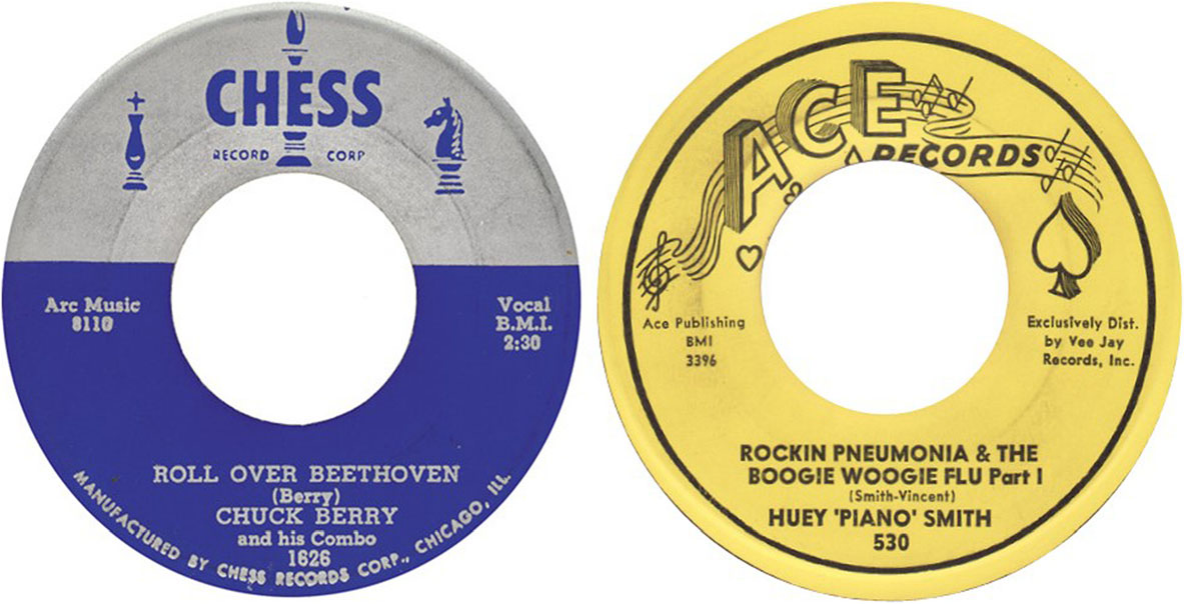
Chuck Berry’s ‘Roll over Beethoven’ and Huey ‘Piano’ Smith’s ‘Rockin’ Pneumonia & the Boogie Woogie Flu’

As indicated above, a number of songs have been published since Chuck Berry’s “Roll over Beethoven”, dealing with many aspects of pneumonia. Below, we analyze lyrics that deal with the cause of pneumonia, risk groups, comorbidity (the boogie woogie flu), the clinical symptoms, and treatment and outcome.

## Cause of pneumonia

In most songs about pneumonia, standing in the rain or in the cold is indicated as cause of the disease. For example, Rod Stewart in “Lost Paraguayos” sings: “it appears to be raining again,. .. honey hurry I'm catching pneumonia” (from the album *Sing It Again Rod*, 1972). The Easybeats in “Come In You’ll Get Pneumonia” sing “Standing in the rain, [.. .] all right, come in you'll get pneumonia*”* (from the album *The Easybeats*, 1981). As recently as 2013, Pusha T in his song ‘Amen’ (from the album *Still Ya Pusha*) sings/raps “And you might get pneumonia, I'm colder than an elf on a sleigh”. The last example is Elvis Presley, from the movie *GI Blues* the song “Didja’ Ever” (from the album *G. I. Blues* 1960), who sings, “Ya get up in the morning and turn the shower on, you're gettin’ pneumonia, the hot water is gone”.

In fact, there is only one song in which an infectious nature of pneumonia is described, “I'll Never Fall in Love Again”, with the lyrics: “What do you get when you kiss a girl? You get enough germs to catch pneumonia”, originally composed by Burt Bacharach in 1969. At the time he composed the song, Bacharach himself was in hospital with pneumonia. There are many different artists who have recorded this classical song. Among them, Dionne Warwick who sings, as all female interpreters do: “What do you get when you kiss a guy?” All male singers kiss a girl. In none of the versions is there a single same sex relation. We can debate on the mode of transmission implied in the lyrics. True, *S. pneumoniae* is readily detectable in saliva. Wyllie et al., using molecular techniques, found 88% of primary school children tested positive for *S. pneumoniae* in saliva [4]. In 1932, Gundel and Okura, using conventional culture techniques, reported 66% of saliva samples from teenagers to be positive for *S. pneumoniae* with point prevalence as high as 85% for boys and 71% for girls [5]. The actual volume of saliva exchanged during intimate kissing is less than 0.5 ml, but it can contain up to 80 × 10^6^ (total) bacteria [6]. Still, unlike the Epstein Barr virus, kissing as route of transmission hasn’t been firmly established for *S. pneumoniae*.

## Risk groups

Pneumonia can affect people of all ages, but the age groups with highest risk of infection are infants below 2 years of age, and people over 65. This is reflected in popular music, with the lyrics in “Streets of New York” from Wolfe Tones describing one of the risk groups when they sing, “little baby daughter … has pneumonia”. Both “Angel of Mercy” from Black Label Society (2014) and “Speed Break” from Abscess also refer to this age group (infants below 2 years of age) as having pneumonia. The older age category is described in “Swing ‘em High” from The Tiger Lillies (1999) “and the old lady’s pneumonia did rage”. Unlike causative agents, as far as risk groups are concerned, these song lyrics accurately describe a fact about pneumonia.

## Comorbidities

The intricate association between pneumonia and influenza is mentioned in a number of songs. The classical “Rocking pneumonia and the boogie woogie flu”, by Huey Smith already alludes to this association. Also Otis Redding in his 1966 song “Hawg for You” sings, “I got rockin’ pneumonia, asiatic flu, I got something to tell you baby!” The Squirrel Nut Zippers in their song ‘La Grippe’ (from the album *The Inevitable*, 1995) also sing, “There's an Asian influenza, infecting us all by the scores, and it's turning into pneumonia”. In more recent songs, in fact since the onset of the AIDS epidemic, pneumonia as a manifestation of an underlying HIV infection is also indicated. This is seen in the song “The Kids” by Eminem (from the album *The Marshall Mathers LP*, 2000) where he sings, “Mr. Kaniff is out with pneumonia (HE’S GOT AIDS!)”.

## Clinical symptoms

The clinical symptoms of pneumonia (high temperature, pleuritic pain and a dry cough, next to shortness of breath, fatigue, confusion, hypothermia, nausea, vomiting or diarrhea) are not at all adequately described in song lyrics. Lightnin’ Hopkins in “Pneumonia Blues” (1964) sings that his “head hurt me so bad till I'm almost to go blind”. Although a few cases have been described in the medical literature, blindness as a complication is a rare event [7, 8].

One of the prominent clinical signs of pneumonia, fever, is rarely mentioned in songs about pneumonia. Danko Jones, in his 1999 song “My love is bold” sings, “I got a fever baby's givin’ me pneumonia”. He is implying that the fever is the cause of the pneumonia, rather than a consequence.

Just a few songs have the single word ‘pneumonia’ as a title. For the purposes of this discussion, which focuses on lyrics, we can ignore “Pneumonia” by Kool and the Gang, because that is an instrumental. Perhaps the most intriguing is Joe Tex’s song entitled “Pneumonia”. In his early career, Joe Tex claimed he was the author of another song, “Fever”, but that he sold it for 300 dollars. According to the label, “Fever” was written by Otis Blackwell in 1956 and first recorded by Little Willie John but made famous by Peggy Lee, Etta James, Madonna, Beyoncé, the Black Keys, and many others. Joe Tex was never recognized as the writer, but 2 years after “Fever” was released, he published his song “Pneumonia”. The melody bears strong resemblance to “Fever”, and even the lyrics are related (see Table 1). At the place where you would intuitively sing “Fever”, he sings “Pneumonia”. There never has been a court case for plagiarism, so maybe Joe Tex actually did write “Fever”. A direct comparison of the lyrics of “Pneumonia” and “Fever” (Table 1) shows how close the relation between pneumonia and fever is.

**Table 1:** Comparison of the lyrics of Pneumonia (Joe Tex) with Fever (Peggy Lee)

Pneumonia	Fever
Oh baby you know that I never did need you	Never know how much I love you
You know that I never did care	Never know how much I care
And if you put your cotton picking arms around me	When you put your arms around me
I’m gonna hit you with this rocking chair	I get a fever that’s so hard to bear
You give me. . pneumonia	You give me. . . fever when you kiss me
Yes, you give me pneumonia baby	Fever when you hold me tight
You know the love you gave me has grown so cold	Fever in the mornin’
I’ve got pneumonia in my heart and soul	A fever all through the night

## Treatment and outcome

In most songs, pneumonia is described as a serious disease for which no treatment is available. In the 1949 musical romantic comedy “Neptune’s Daughter”, made during a time when antibiotics were not yet generally available, Ricardo Montalbán and Esther Williams sing the Academy Award winning song “Baby it’s cold outside”, which includes the line, “If you got pneumonia and died*”*. The only intervention that could help is a shot of rhythm and blues: “I got a rocking pneumonia, I need a shot of rhythm and blues”. Other treatment options put forward in songs about pneumonia are even less evidence-based; for example, treatments such as “soy bologna”, according to Nellie McKay’s song “Suitcase Song” (2004), and “prayers” from Rod Stewart’s ‘Lost Paraguayos’.

In the song “Lost Weekend” (2004), Lloyd Cole mentions treatment but complains about the costs of antibiotics (“It took a lost weekend in a hotel in Amsterdam, and double pneumonia in a single room, and the sickest joke was the price of the medicine”). Lloyd Cole must have travelled on a tight budget, because the price of a course of amoxicillin is well below €60, less than a single night in a cheap Amsterdam hotel. This shows us that it took almost half a century of pneumonia being mentioned in song lyrics before the ability to treat pneumonia with antibiotics also found its way into popular music. We may have to wait another 50 years before songwriters will realize that pneumonia can be prevented by vaccination, rather than by an umbrella, or refraining from kissing girls.

## Editor-in-Chief’s challenge

Rather than waiting for current knowledge of vaccine efficacy to filter down to popular culture, perhaps the next step in raising awareness about pneumonia is to take our knowledge of the disease and pen a song about pneumonia prevention—who knows, it might just be a hit!

Thus, the *Pneumonia* journal has a holiday challenge for you: to compose lyrics for a song that highlights vaccination as a means of preventing pneumonia. The most fitting submissions will be published as responses to this commentary.

## References

[1] Woolf V. On being ill. In: Woolf Online. http://www.woolfonline.com/?node=content/contextual/transcriptions&project=1&parent=56&taxa=45&content=6225&pos=13. Accessed 7 Oct 2017.

[2] https://youtu.be/53rRTwRwxcg. Accessed 9 Oct 2017.

[3] Wirth J (2014). Huey ‘piano’ smith and the rocking pneumonia blues.

[4] Wyllie AL, Chu ML, Schellens MH, van Engelsdorp GJ, Jansen MD, van der Ende A (2014). *Streptococcus pneumoniae* in saliva of Dutch primary school children. PLoS One.

[5] Okura G. Untersuchungen über das gleichzeitige Vorkommen mehrerer Pneumokokkentypen bei Gesunden und ihre Bedeutung für die Epidemiologie [in German]. Zeitschrift für Hyg und Infekt. 1933;114:678–704.

[6] Kort R, Caspers M, van de Graaf A, van Egmond W, Keijser B, Roeselers G (2014). Shaping the oral microbiota through intimate kissing. Microbiome.

[7] Garcia Tirado A, Jimenez-Rolando B, Noval S, Martinez BA (2017). Cortical blindness in a child secondary to *Mycoplasma pneumoniae* infection. J Stroke Cerebrovasc Dis.

[8] Nussbaumer-Ochsner Y, Hasse BK, Valmaggia C, Krause M. A pneumonia leading to blindness. BMJ Case Rep. 2015; doi: 10.1136/bcr-2014-208749. 10.1136/bcr-2014-208749 PMC4434312 25948849

